# Relationship of Thermal Treatment and Antioxidant Capacity in Cooked Foods

**DOI:** 10.3390/antiox11122324

**Published:** 2022-11-24

**Authors:** Beatriz Navajas-Porras, Sergio Pérez-Burillo, Daniel Hinojosa-Nogueira, Silvia Pastoriza, José Ángel Rufián-Henares

**Affiliations:** 1 Departamento de Nutrición y Bromatología, Instituto de Nutrición y Tecnología de Alimentos, Centro de Investigación Biomédica, Universidad de Granada, 18071 Granada, Spain; 2 Instituto de Investigación Biosanitaria ibs.GRANADA, Universidad de Granada, 18071 Granada, Spain

**Keywords:** maillard reaction, furosine, HMF, furfural, cooking, antioxidant activity

## Abstract

Most of the foods we eat undergo a cooking process before they are eaten. During such a process, the non-enzymatic browning occurs, which generates compounds such as furosine, 5-hydroxymethylfurfural (HMF) and furfural. These are considered markers of cookedness and can therefore be used as quality indicators. In this work, we study the production of these compounds in different foods (both of plant and animal origin) that are cooked with different techniques. Additionally, we investigate correlations between the production of these markers of cookedness and the antioxidant capacity produced after in vitro digestion and fermentation. We observe that, in general, cereals and vegetables are more thermally damaged. Toasting and frying produce the highest concentrations of Maillard compounds whereas boiling the lowest. Furosine content shows a significant positive correlation with in vitro digestion data in fried foods, and with fermentation in roasted foods. Furfural content shows a significant positive correlation with in vitro digestion results in roasted foods, specifically in the Folin–Ciocalteu method.

## 1. Introduction

Cooking a food is an operation that can modify its characteristics to improve the organoleptic properties, digestibility and hygienic conditions. In addition, cooking most frequently involves a heat source in order to raise its temperature. As consequence, food undergoes physical, chemical, or biological changes. Although cooking usually improves taste, flavor or make food safe to be consumed, it can also have a negative impact on the food chemical composition and by extension on human health [1]. There are several types of cooking that can be classified according to how heat is transmitted onto the food. For example, frying or grilling uses fats as the medium to transfer heat to the food [2] whereas boiling uses water. On the other hand, others (like roasting) use the air to transfer heat to the food surface.

Heating favors a plethora of chemical changes within the food, and some of those as consequence of the non-enzymatic browning, including the Maillard reaction [3]. This reaction involves a set of chemical chain reactions that is favored when food is subjected to moderate heat and gives rise to a plethora of molecules responsible for new colors, smells, tastes and textures that are usually pleasing to the consumer [4,5], although undesirable aromatic substances and brown compounds may also be produced [6]. In order to allow the reaction takes place, a free carbonyl group is needed (such as those from reducing sugars, oxidized lipids or B group vitamins) as well as free amino groups from an amino acid, peptide or protein [7]. The Maillard reaction is divided into three stages; during the early stage, while it is still reversible and browning has not yet occurred, sugars and amino acids begin to degrade [8,9]. Furosine appears during this stage [10]. It was one of the first products to be identified for the Maillard reaction. The concentration of this compound has been shown to increase as a function of the heat treatment applied and is another marker of heat damage [11,12]. Secondly, the intermediate stage involves dehydration of sugars by enolic isomerization, giving rise to furfural and 5-hydroxymethylfurfural (HMF) among other compounds. Furfural content of foods correlates with undesirable flavors and is therefore a good quality indicator [13]. HMF also allows one to monitor intermediate stages of the Maillard reaction and it is an indicator commonly used by the food industry to assess heat damage in plant food products [14,15]. The final stage involves polymerization and formation of high molecular weight-colored substances called melanoidins [16].

It is remarkable that water-soluble compounds generated during MR have shown the ability to neutralize free radicals [17]. Such antioxidant capacity is proportional to the degree of browning [18] and has a close correlation with the compounds generated from the intermediate and late stages, as well as with the type of sugar involved in the reaction [19]. Despite the partial loss of natural compounds with antioxidant activity that may occur during food processing, antioxidant properties could be maintained and even increased due to the formation of new compounds through the development of the Maillard reaction [20] or release by cell breakage [21]. In previous studies, we found that cooking techniques strongly modify the antioxidant capacity of plant [22] and animal foods [23]. Therefore, the aim of this study was to unravel the potential contribution of the development of non-enzymatic browning to the antioxidant capacity of foods. To do that, 23 of the most commonly consumed foods in Spain were submitted to common cooking techniques (including frying, roasting, toasting, boiling and grilling). Furosine, HMF and furfural concentrations were analyzed as indicators of non-enzymatic browning, related with the cookedness of foods. In addition, correlation studies were carried out between these indicators and antioxidant capacity in the same foods with the same cooking, both after in vitro digestion and colonic fermentation stages.

## 2. Materials and Methods

### 2.1. Chemicals

Furosine was purchased from NeoMPS (Strasbourg, France). Furfural, 5- (hydroxymethyl)furfural, hydrochloric acid, methanol, and acetonitrile (HPLC grade) were obtained from Sigma-Aldrich (Taufkirchen, Germany). Alpha Aesar provided the pancreatin in the porcine pancreas (Heysham, UK). The remaining chemicals, which included analytical-grade salts and enzymes for in vitro digestion and fermentation as well as chemicals and solvents for the determination of antioxidant capacity, were bought from Sigma-Aldrich (Taufkirchen, Germany).

### 2.2. Foods and Cooking Conditions

A total of 20 foods were studied, and included in these groups were: cereals (bread, bread whole grain, penne, penne whole grain, rice, rice whole grain), egg, fish (cod fish and salmon), fruits (apple and banana), legumes (beans and lentils), meat (pork, beef, chicken and lamb), tubers (potato) and vegetables (capsicum, carrot, cauliflower, onion and tomato). Different thermal processes were applied to the samples: boiling, frying, grilling, roasting and toasting) (Table S1). Fruits, tubers and vegetables were cut into different sizes so that the same texture was achieved after the different cooking processes (See Table S1).

For grilling and frying, extra virgin olive oil (EVOO) was used as a cooking medium. Boiling was performed at a water/food rate of 5:1, for 20 min at 100 °C. Grilling was carried out at an oil/food rate of 0.5:1, for 3 min on each side, at 220–250 °C. Fried foods were obtained at an oil/food rate of 5:1, at 180 °C for 8 min. Roasting was performed for 10 min at 180 °C. Toasting was carried out for 3 min at 900 W, in a Grunkel TS140H toaster at the fourth level following the manufacturer’s instructions. Cooking times and food/average rates were acquired from previous work [2].

The utensils used for foods preparation were forks and knives, stainless steel spoons; frying pan, saucepan, fryer, a portable oven (1500 W), and toaster. These utensils were acquired at Centro Hogar Sánchez (Granada, Spain). Cooked foods were homogenized and stored at −80 °C under a nitrogen atmosphere. All analyses were performed in duplicate.

### 2.3. Furosine, HMF and Furfural Assays

Furosine assay was carried out following the method of Delgado-Andrade et al. [24]. Samples were hydrolyzed for 23 h at 120 °C with 7.95 M HCL. The hydrolysate was purified with a Sep-pack C_18_ cartridge (Millipore, Burlinton, MA, USA), and the resulting solution was analyzed by ion pair RP-HPLC. The analysis was performed in duplicate, and the data are mean values expressed as μg/g food and μg/g of protein. Protein in each food was estimated from a database [25].

HMF and furfural were determined following a previously described protocol [14]. Ground samples were suspended in deionized water, clarified with Carrez I (potassium ferrocyanide, 15% *w*/*v*) and Carrez II (zinc acetate 30% *w*/*v*) solutions. The resulting solution was analyzed by RP-HPLC. The analysis was performed in duplicate, and the data are mean values expressed in μg per g of food.

### 2.4. In Vitro Gastrointestinal Digestion and Fermentation and Antioxidant Capacity

All samples were submitted to in vitro digestion-fermentation according to the protocol previously described [26,27]. Five g of each food was subjected to in vitro gastrointestinal digestion followed by in vitro fermentation, in triplicate. The in vitro fermentation was carried out using fecal material from five healthy donors (with a mean Body Mass Index = 21.3, and who had not taken antibiotics for three months prior to the assay). All fecal samples were pooled together to restricted inter-individual variability. The fermentation was carried out for 24 h, at 37 °C. After the in vitro gastrointestinal digestion and fermentation, two fractions were obtained: digestion supernatant, which is available for absorption at the small intestine, and fermentation supernatant, which is available for absorption at the large intestine. A control fermentation was carried out, using only the fecal fermentation solution (inoculum composed of peptone, cysteine, and resazurin).

The antioxidant capacity was evaluated in the two supernatant fractions obtained after in vitro digestion and fermentation, which represent the total antioxidant capacity that each food could exert in the human body [28]. Three different methods were used to determine the antioxidant capacity (DPPH, FRAP and Folin–Ciocalteu). The results of such analyses were described in previous work for plant [22] and animal foods [23].

### 2.5. Statistical Analysis

Statistical differences were calculated using the unpaired Kruskal Wallis test with 95% confidence, comparing the amount of furosine, HMF and furfural in each of the food groups, as well as within each group, and the comparison was made by cooking. Thus, we show whether a particular food group has a higher or lower amount of these indicators of cookedness. Pearson’s parametric statistic was calculated to show the linear relationship between the heat damage markers and between these and the antioxidant capacity produced in the same foods with the same thermal processing at *p*-value < 0.05. Correlations were made for both antioxidant capacity after in vitro digestion of foods and after in vitro fermentation with healthy adult microbiota. The correlations were based on the cooking methods used for the different food groups. The Statgraphics Plus software (version 5.1) was used to perform all statistical analyses.

## 3. Results

### 3.1. Furosine Content of Cooked Foods

**Figure 1:**
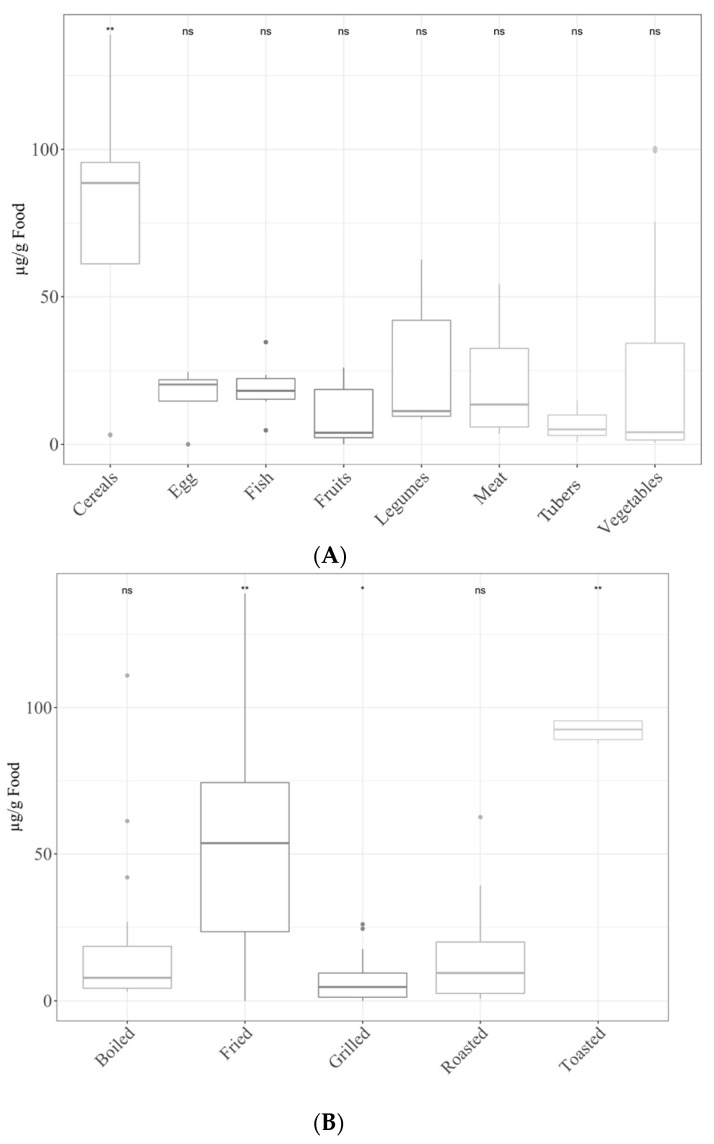
(**A**) Furosine levels in different food groups. Statical analysis was performed via Kruskal–Wallis test. Each of the groups were compared to the average of all of them (i.e., base-mean). Statistic labels: *: *p* < 0.05. **: *p* < 0.01, ns: not significant. (**B**) Furosine levels depending on the cooking applied. Statical analysis was performed via Kruskal–Wallis test. Each group was compared to the average of all of them (i.e., base-mean). Statistic labels: *: *p* < 0.05, **: *p* < 0.01, ns: not significant.

For the furosine assay, the food group with the highest concentration after thermal processing was cereals, followed by vegetables, meat, legumes, fish, eggs, fruits and tubers. The values for cereals were significantly (*p* < 0.05) higher than the mean of the other food groups (Figure 1A). When we consider cooking techniques, toasting and frying gave the highest (*p* < 0.05) concentrations. Grilling, on the other hand, showed significantly (*p* < 0.05) lower concentrations than the rest (Figure 1B).

#### Furosine Content by Specific Foods and Cooking Methods

Table 1 shows the furosine content of each food depending on the kind of thermal treatment used for cooking. In the cereals group, the highest furosine value was reached with frying (138.9 μg/g), while in the case of eggs it was obtained for grilled eggs (24.5 μg/g). In the fish group, again fried foods had the highest furosine content (fried salmon, 34.6 μg/g) but for fruits, was boiled banana (42.0 μg/g). In legumes, roasted kidney beans demonstrated a high reactivity (furosine values of 62.6 μg/g), but were meat and vegetables the groups with the highest levels of furosine: 412.5 μg/g for fried cauliflower and 183.6 μg/g for fried meat. Thus, in general, frying (followed by roasting) was the cooking method that produced the highest levels of furosine. Table S2 shows the correlations depending on the food group.

Furosine is a good indicator of the thermal damage suffered by proteins during heat treatment [3,5,10] since it is correlates with the loss of available lysine. Thus, furosine can be also expressed in mg/100 g of protein to show the thermal damage (or heat load) of the food. In this sense, the highest thermal damage was suffered by vegetables (fried cauliflower and carrot with values surrounding 800 mg furosine/100 g of protein) closely followed by fruits (fried banana with a value of 620 mg furosine/100 g of protein) and cereals (fried bread, 163.4 mg furosine/100 g of protein). Again, frying was the heat treatment with the highest thermal damage, while boiling and grilling were the milder cooking option.

### 3.2. HMF Content of Cooked Foods

**Figure 2:**
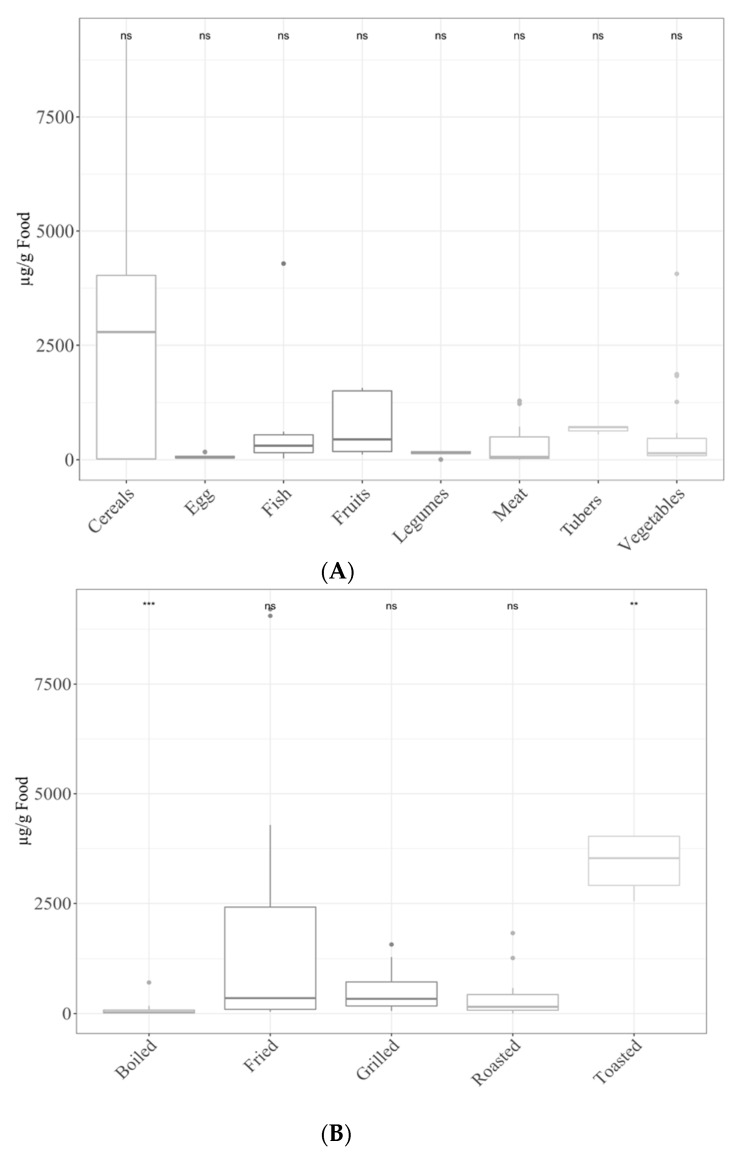
(**A**) HMF levels in different food groups. Statical analysis was performed via Kruskal–Wallis test. Each of the groups were compared to the average of all of them (i.e., base-mean). Statistic labels: ns: not significant. (**B**) HMF levels depending on the cooking applied. Statical analysis was performed via Kruskal–Wallis test. Each group was compared to the average of all of them (i.e., base-mean). Statistic labels: **: *p* < 0.01, ***: *p* < 0.001, ns: not significant.

Regarding HMF content, the food group that presented the highest amount of this compound after the different thermal processes was cereals, followed by vegetables, fish, fruit, tubers, meat, legumes and eggs (Figure 2A). As for cooking techniques, toasting generated the highest concentrations (*p* < 0.05). Frying, roasting, grilling and boiling followed toasting in HMF production, though only boiling produced significantly lower levels (*p* < 0.05) than the rest (Figure 2B).

#### HMF Content by Specific Foods and Cooking Methods

The levels of HMF in each food (Table 2) were also used to study the effect of the cooking method on the development of non-enzymatic browning. As expected, the heat treatment of cereals produced the highest HMF concentration, up to 10,305 μg/g for toasted bread and five times lower for fried bread. Eggs and legumes where not too much affected by cooking, with grilling the most damaging cooking method (169 and 179 μg/g for grilled egg and lentils). Meats showed a higher content than fish after cooking, with being grilling again being the most harmful thermal treatment (1287 and 613 μg/g for pork and salmon, respectively). In addition, cooking potatoes generated large amounts of HMF, ranging from 550 μg/g during frying till 737 μg/g after grilling. On the other hand, fruits and vegetables were highly reactive during cooking, showing high HMF levels for grilled banana and fried apple (around 1500 μg/g) and close to 4000 and 2000 μg/g for fried onion and cauliflower, respectively (Table 2). Opposite to furosine, in the case of HMF generation, there was not a single cooking method with a higher thermal damage, since frying, grilling, roasting and toasting produce large amounts of HMF, depending on the food matrix. Table S2 shows the correlations depending on the food group.

### 3.3. Furfural Content of Cooked Foods

**Figure 3:**
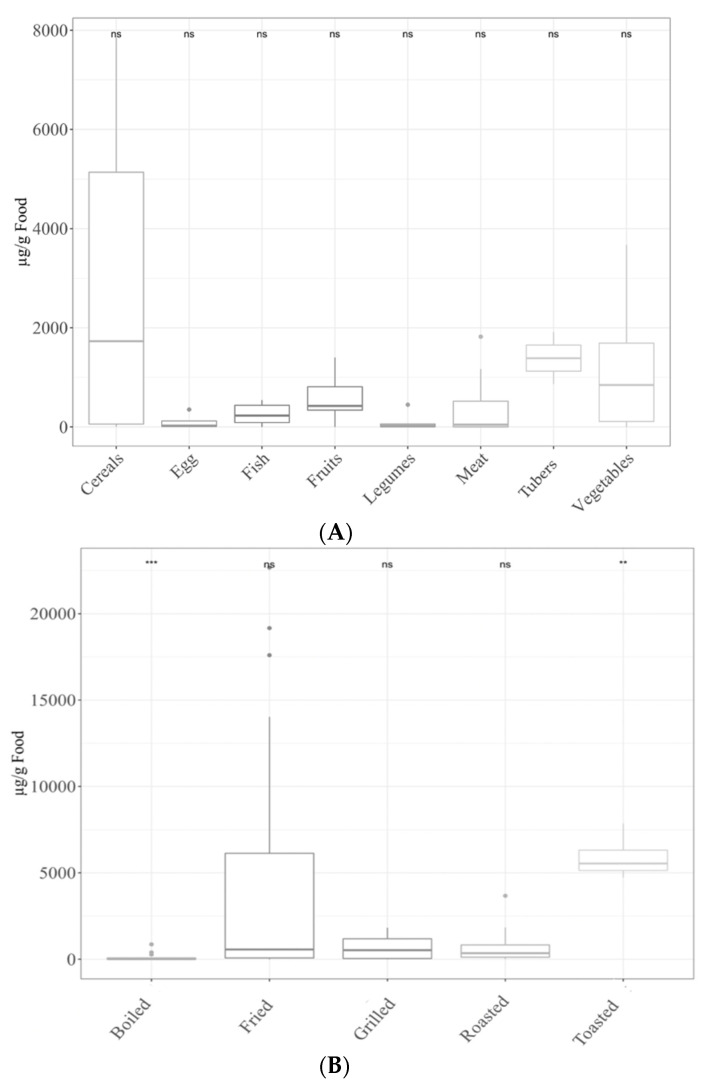
(**A**) Furfural levels in different food groups. Statical analysis was performed via Kruskal–Wallis test. Each of the groups were compared to the average of all of them (i.e., base-mean). Statistic labels: ns: not significant. (**B**) Furfural levels depending on the cooking applied. Statical analysis was performed via Kruskal–Wallis test. Each group was compared to the average of all of them (i.e., base-mean). Statistic labels: **: *p* < 0.01, ***: *p* < 0.001, ns: not significant.

Regarding furfural, tubers showed the largest levels (*p* < 0.05), followed by cereals, fruits, vegetables, meat, fish, legumes and eggs (Figure 3A). Toasting generated the highest concentrations (*p* < 0.05). After toasting, in decreasing order of furfural content, we found frying, grilling, roasting and finally boiling. Only the latter showed significantly (*p* < 0.05) lower concentrations than the average of the rest (Figure 3B).

#### Furfural Content by Specific Foods and Cooking Methods

Furfural was another furanic compound used as an indicator of thermal treatment (Table 3). Boiling produced low levels of furfural in cereals such as penne or rice, but roasted and fried bread generated large amounts of this furanic compound (7859 and 1192 μg/g, respectively). In the case of protein-rich foods, eggs and salmon had a relatively high furfural content (from 352 till 545 μg/g), but fried meats (pork and chicken) were those with higher values (over 1100 μg/g in both cases). The highest furfural levels were obtained in cooked tubers and vegetables, reaching very high furfural values: around 17,600 and 19,200 μg/g for fried onion and potatoes, respectively (Table 3). As in the case of HMF, there was not a single cooking method producing the largest furfural contents, since frying, grilling, roasting and toasting produced high furfural levels depending on the food. It is noteworthy to mention that boiling was the less aggressive heat treatment, giving rise to low furfural levels or even not detected in meats and legumes. Table S2 shows the correlations depending on the food group.

### 3.4. Correlation of Heat Damage Markers with Antioxidant Capacity of Cooking Foods after Digestion and Fermentation

**Figure 4:**
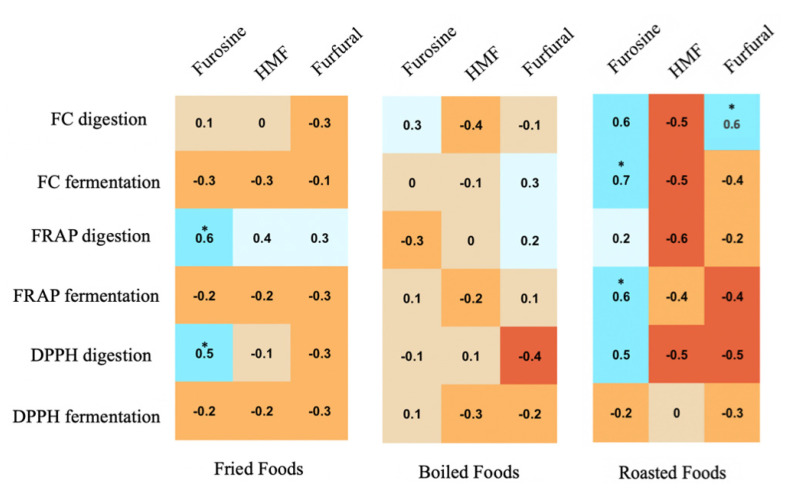

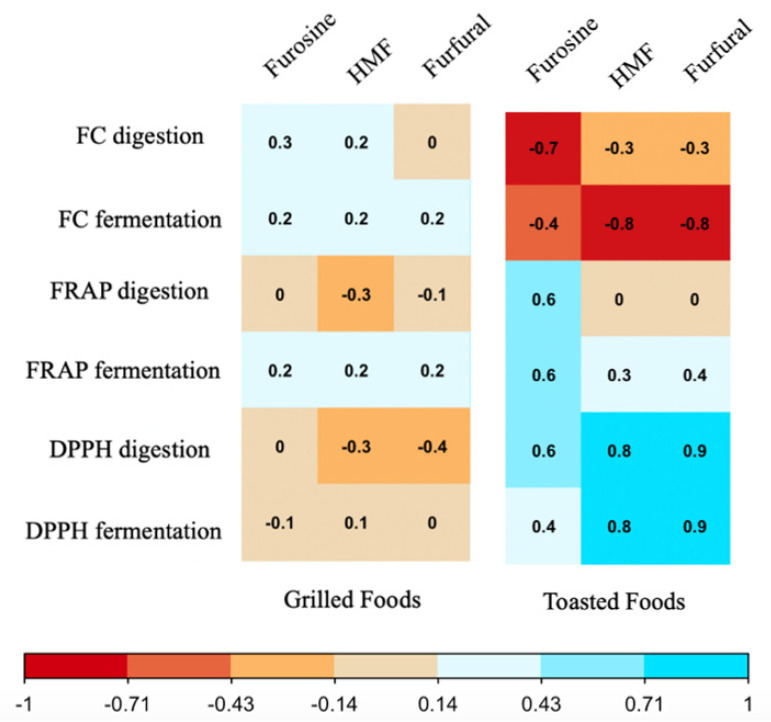
Correlations between heat damage markers (furosine, HMF and furfural) and antioxidant capacity measured via Folin–Ciocalteu (FC) (mg gallic acid equivalents/kg of food), FRAP (mmol Trolox equivalents/kg of food) and DPPH (mmol Trolox equivalents/kg of food) depending on the type of cooking applied. Statistic labels: *: *p* < 0.05.

The results of antioxidant capacity are shown in Table 4 and are deeply described in previous work for plant [22] and animal foods [23]. In general, it was found that intense cooking methods, such as frying, increase the antioxidant capacity of foods. In the case of animal foods, meat was the group with the highest antioxidant capacity [23], while cocoa and legumes were the most antioxidant plant foods [22]. Taking all this information into account, we generated correlations between heat damage markers (furosine, HMF and furfural) and antioxidant capacity obtained after in vitro digestion and fermentation (Figure 4).

Fried foods: Furosine correlated positively with Folin–Ciocalteu, FRAP and DPPH obtained after in vitro digestion, though only the last two were statistically significant (*p* < 0.05). HMF and furfural content correlated negatively with all antioxidant capacity results obtained, except with FRAP for digestion (Figure 4).

Boiled foods: Although there were no statistically significant correlations, furosine content was positively correlated with Folin–Ciocalteu results for digestion, and FRAP and DPPH for fermentation (Figure 4). Furosine correlated negatively with the rest. In the case of HMF content, it was correlated negatively with all antioxidant capacity results except for DPPH obtained from the digestion fraction. Finally, furfural content correlated positively with antioxidant capacity results obtained via Folin–Ciocalteu for fermentation and FRAP for both digestion and fermentation. The rest of the correlations were negative.

Roasted foods: Furosine correlated positively and significantly (*p* < 0.05) with Folin–Ciocalteu, FRAP for fermentation, and the correlation was statistically significant. It was also positively correlated with Folin–Ciocalteu, FRAP and DPPH for digestion, and negatively correlated with DPPH for fermentation (Figure 4). HMF content was negatively correlated with all antioxidant capacity results. As for furfural content, it correlated negatively with all results except with Folin–Ciocalteu for digestion, which correlated positively and, moreover, in a statistically significant manner (*p* < 0.05).

Grilled foods: No statistically significant correlations were found, but furosine content was positively correlated with Folin–Ciocalteu for digestion and fermentation, FRAP for fermentation and DPPH for digestion (Figure 4). With the others it correlated negatively. HMF content was positively correlated with Folin–Ciocalteu results for digestion and fermentation and FRAP and DPPH for fermentation. The rest of the correlations were negative. Furfural content for grilled foods was positively correlated with all antioxidant capacity results except FRAP and DPPH for digestion, with which it was negatively correlated.

Toasted foods: Furosine content correlated negatively with Folin–Ciocalteu antioxidant capacity results for digestion and fermentation, although FRAP and DPPH in both digestion and fermentation correlated positively. The same was true for HMF and furfural content.

## 4. Discussion

Non-enzymatic browning products present both benefits [29,30,31] and risks [32,33] and research must find a balance between both of them [1]. Furosine is formed along the Maillard reaction from Amadori compounds, and originates before sensory changes take place. Therefore, it is considered a very sensitive indicator for the early detection of quality changes [34]. HMF and furfural are formed both at the intermediate stages of the Maillard reaction and also under acidic conditions involve degradation of sugars at high temperature, known as caramelization, indicating a higher degree of cookedness [14,35]. So, these furanic compounds are indicators of non-enzymatic browning.

In our assays, cereals and vegetables stood out for their high furosine and HMF content. Particularly, cauliflower and bread showed the highest levels. On the other hand, tubers and cereals showed the highest concentration of furfural, particularly bread, agreeing with furosine and HMF levels. These results are in agreement with other previous studies [1] where cereals and tubers in combined dishes considerably increase the amount of furosine, HMF and furfural. Specifically, bread is a food that produces high levels of furosine due to its composition [36]. Eggs exhibited the lowest values of the three indicators, which may be due to a lower level of sugars and amino acids ready to react [1,36,37].

Toasted and fried foods showed higher levels of furosine, HMF and furfural than the rest. Previous studies have shown that toasting and frying generate a considerable concentration of cookedness markers [1,31], and even different nutritional studies advise against the consumption of toasted foods [32]. Cooking plays an important role in the appearance of Maillard reaction products; both the time and the temperature applied are decisive. In the case of frying, in addition to the heat applied, the composition of the frying oil plays a role, in which, compounds derived from oil decomposition can be formed [38,39,40]. The cooking that contributed the least amount of HMF and furfural to the food was boiling, being furfural not detected in many boiled foods (beans, lentils, chicken, beef, pork, lamb and tomatoes). This may be due to the temperature used for boiling (100 °C) which is a low temperature compared to other cooking temperatures, including also a dilution effect of the boiling water, which impede the reaction among carbohydrates and amino acids. Grilling was carried out at a higher temperature (250 °C), but for a very short time, including also a small amount of oil. Frying was carried out at a lower temperature (180 °C) but for a longer time, and the amount of oil used was much higher than in grilling. Finally, toasting was only carried out for 3 minutes, but at very high intensity and on foods rich in sugars. All these cooking characteristics could explain the results found. 

In addition to the content of furosine, HMF and furfural, in this work we studied the correlations between the indicators of cookedness in foods with the antioxidant capacity of the same foods, once digested and fermented in vitro. We mainly focused on the classification according to the type of cooking applied (Figure 4), but correlations were also carried out taking into account the different food groups (Table S2). Figure 4 shows that statistically significant correlations were positive: Furosine content and antioxidant capacity obtained from FRAP and DPPH digestion for fried foods, furosine content and antioxidant capacity obtained from Folin-Ciocalteu and FRAP digestion, and furfural content with the antioxidant capacity obtained from the Folin-Ciocalteu assay of digested foods for the roasted treatment. All these correlations could argue that aggressive treatment (such as frying and roasting) could improve the availability of some molecules to react through non-enzymatic browning [1,40,41], while the lack of correlations in milder cooking techniques (i.e., boiling or grilling) could indicate that cellular breakdown and reactants release could be the responsible in antioxidant differences [15,22,23]. Such positive correlations have also been described in other studies. Seiquer et al. [42] reported that a diet rich in processed foods (with a high concentration of Maillard reaction products) was able to suppress lipid peroxidation and increase plasma antioxidant activity, but without modifying the antioxidant activity of enzymes (superoxide dismutase, glutathione peroxidase and catalase). In another study carried out by Manzocco et al. [43], the data on color changes due to browning reactions and their relationship with the formation of antioxidant capacity components were analyzed, finding that the Maillard reaction promotes positive variations in the antioxidant properties of foods, which are directly proportional to the development of browning. Furthermore, a linear correlation (R^2^) was found with values of 0.80–0.99, which confirms the positive correlation between color and antioxidant activities. In another study, the antioxidant capacity of barley increased with heating intensity, in parallel with color formation [44]. The baking process of barley malt could induce the formation of water-soluble Maillard reaction products, which exerted a strong free radical scavenging capacity [45].

## 5. Conclusions

This study has shown that foods with a high sugar content (such as bread or some vegetables) generate more Maillard reaction compounds than foods whose composition is mainly proteical (such as eggs or lamb). Another point to consider is the cooking technique applied, in particular roasting and frying induced a stronger thermal damage and a higher development of non-enzymatic browning. The Maillard reaction produces sensory changes in the food that are considered positive (e.g., taste or smell). Thus, in this case it is important to consider the production of furosine, HMF and furfural as good indicators of cookedness, even when the organoleptic characteristics of the food have not yet changed (as in the case of furosine). As for the correlations of the indicators of cookedness with antioxidant capacity, the furosine and furfural content stood out for their statistically positive correlations with the antioxidant capacity of the samples in fried and roasted foods.

## Figures and Tables

**Table 1:** Furosine values depending on the cooking method applied to the food.

Food Group	Food	Boiled		Fried		Grilled		Roasted		Toasted	
		μg/g Food	mg/100 g Protein	μg/g Food	mg/100 g Protein	μg/g Food	mg/100 g Protein	μg/g Food	mg/100 g Protein	μg/g Food	mg/100 g Protein
Cereals	Bread	-	-	138.9	163.4	-	-	-	-	95.4	127.2
	Penne	3.1	5.8	-	-	-	-	-	-	-	-
	Rice	19.5	84.8	-	-	-	-	-	-	-	-
Egg	Egg	14.5	7.1	n.d.	n.d.	24.5	11.5	21.0	8.6	-	-
Fish	Cod fish	-	-	23.5	8.9	4.7	2.6	-	-	-	-
	Salmon	-	-	34.6	8.9	17.6	7.9	18.6	7.2	-	-
Fruits	Apple	-	-	18.6	620.0	n.d.	n.d.	2.2	73.3	-	-
	Banana	42.0	323.1	-	-	26.0	123.8	3.9	32.5	-	-
Legumes	Beans (Kidney)	8.5	14.9	-	-	-	-	62.6	88.2	-	-
	Lentils	40.5	61.4	-	-	11.2	14.7	9.5	11.7	-	-
Meat	Beef	72.4	29.7	183.6	67.0	5.6	1.9	39.2	19.2	-	-
	Chicken	26.8	9.6	54.2	18.4	7.7	3.0	28.9	12.6	-	-
	Pork	59.6	22.7	30.2	9.7	3.6	2.0	8.0	3.7	-	-
	Lamb	-	-	45.4	18.5	5.4	3.6	19.0	11.7	-	-
Tubers	Potatoe	5.0	0.010	14.8	0.062	0.9	0.002	-	-	-	-
Vegetables	Capsicum	3.4	37.8	53.2	110.8	0.5	10.0	1.4	15.6	-	-
	Carrot	14.6	162.2	99.4	764.6	4.1	41.0	0.9	5.6	-	-
	Cauliflower	6.5	31.0	412.5	808.8	1.5	7.5	13.7	52.7	-	-
	Onion	3.9	32.5	74.0	528.6	0.8	5.7	2.8	4.4	-	-

n.d. = not detected. The sign—denotes that such cooking method was not used for that particular food.

**Table 2:** HMF values (expressed in μg HMF/g of food) depending on the cooking method applied to the food.

Food Group	Food	Boiled	Fried	Grilled	Roasted	Toasted
Cereals	Bread	-	2057.0	-	-	10,304.9
	Penne	6.7	-	-	-	-
	Rice	15.3	-	-	-	-
Egg	Egg	47.4	43.8	168.9	26.5	-
Fish	Cod fish	30.8	429.5	336.0	-	-
	Salmon	-	275.2	613.2	115.3	-
Fruits	Apple	-	1505.6	114.1	444.0	-
	Banana	-	n.d.	1572.8	179.4	-
Legumes	Beans (Kidney)	175.8	-	-	154.1	-
	Lentils	5.0	-	178.5	134.5	-
Meat	Beef	13.3	1222.5	720.8	52.9	-
	Chicken	33.5	351.8	720.9	333.0	-
	Pork	4.5	63.7	1286.9	423.3	-
	Lamb	2.6	52.0	61.7	6.2	-
Tubers	Potatoe	708.0	550.4	737.4	-	-
Vegetables	Capsicum	88.0	71.7	352.6	63.2	-
	Carrot	55.1	104.1	223.3	89.5	-
	Cauliflower	48.9	1868.4	89.8	581.1	-
	Onion	127.6	4065.5	237.0	1832.1	-

n.d. = not detected. The sign—denotes that such cooking method was not used for that particular food.

**Table 3:** Furfural values (expressed in μg Furfural/g of food) depending on the cooking method applied to the food.

Figure	Food	Boiled	Fried	Grilled	Roasted	Toasted
Cereals	Bread	-	1191.6	-	-	7858.6
	Penne	47.3	-	-	-	-
	Rice	98.2	-	-	-	-
Egg	Egg	42.9	n.d.	13.1	351.8	-
Fish	Cod fish	268.0	55.5	n.d.	-	-
	Salmon	-	494.3	544.5	191.1	-
Fruits	Apple	-	14,028.8	1.8	810.3	-
	Banana	-		425.4	338.3	-
Legumes	Beans (Kidney)	n.d.		-	449.0	-
	Lentils	n.d.		61.5	27.4	-
Meat	Beef	n.d.	613.6	131.5	90.3	-
	Chicken	n.d.	514.7	1168.0	533.9	-
	Pork	n.d.	54.6	1821.1	41.7	-
	Lamb	n.d.	n.d.	18.5	20.0	-
Tubers	Potatoe	864.2	19,164.0	1386.5	-	-
Vegetables	Capsicum	3.8	232.7	1748.4	1844.1	-
	Carrot	83.7	76.2	844.3	139.7	-
	Cauliflower	3.3	3496.7	1199.2	851.8	-
	Onion	396.4	17,596.9	524.5	3672.6	-

n.d. = not detected. The sign—denotes that such cooking method was not used for that particular food.

**Table 4:** Antioxidant capacity measured via Folin–Ciocalteu (mg gallic acid equivalents/kg of food), FRAP (mmol Trolox equivalents/kg of food) and DPPH (mmol Trolox equivalents/kg of food) depending on the type of cooking applied.

AOX Method	In Vitro Pre-Treatment	Boiled	Fried	Grilled	Roasted	Toasted
Folin-Ciocalteu	Digestion	1259 ± 1144	1368 ± 1018	1409 ± 1374	2262 ± 1609	3536 ± 268
	Fermentation	33,396 ± 12,455	38,221 ± 19,990	43,498 ± 21,926	43,837 ± 18,024	16,573 ± 5625
FRAP	Digestion	2.3 ± 2.0	6.8 ± 5.7	4.1 ± 2.4	4.6 ± 3.0	4.9 ± 1.0
	Fermentation	179 ± 66.9	202 ± 115	239 ± 118	243 ± 98.4	97.4 ± 26.0
DPPH	Digestion	13.2 ± 9.6	22.3 ± 24.1	12.1 ± 8.7	18.0 ± 13.8	1.1 ± 0.8
	Fermentation	213 ± 219	222 ± 206.9	253 ± 199	290 ± 215	108 ± 21.5

## Data Availability

The data presented in this study are available upon request to Prof. José A. Rufián-Henares.

## Supplementary Materials

The following supporting information can be downloaded at: https://www.mdpi.com/article/10.3390/antiox11122324/s1: Supplemental Table S1. Samples: foods and applied thermal processing. Supplemental Table S2. Correlations between heat damage markers (furosine, HMF and furfural), and the results of antioxidant capacity measured with Folin-Ciocalteu, FRAP and DPPH after digestion and in vitro fermentation processes, depending on the different foods groups.
