# Supplementary material for: Relationship of Thermal Treatment and Antioxidant Capacity in Cooked Foods
Source: Antioxidants (Basel). 2022 Nov 24;11(12):2324. doi: 10.3390/antiox11122324 (PMC9774919; doi:10.3390/antiox11122324)
Supplement: Supplementary file 1 [file antioxidants-11-02324-s001.zip › antioxidants-1977298-supplementary.pdf]

# Relationship of thermal treatment and antioxidant capacity in cooked foods

# SUPPLEMENTAL INFORMATION

**Table S1.** Samples: foods and applied thermal processing.

| Food Group | Food | Cooking Method |
|---|---|---|
| Cereals | Bread | Fried |
| Cereals | Bread | Toasted |
| Cereals | Penne | Boiled |
| Cereals | Rice | Boiled |
| Egg | Egg | Boiled |
| Egg | Egg | Grilled |
| Egg | Egg | Fried |
| Egg | Egg | Roasted |
| Fish | Cod fish | Boiled |
| Fish | Cod fish | Grilled |
| Fish | Cod fish | Fried |
| Fish | Salmon | Grilled |
| Fish | Salmon | Fried |
| Fish | Salmon | Roasted |
| Fruits | Apple | Grilled |
| Fruits | Apple | Fried |
| Fruits | Apple | Roasted |
| Fruits | Banana | Grilled |
| Fruits | Banana | Roasted |
| Legumes | Beans (Kidney) | Boiled |
| Legumes | Beans (Kidney) | Roasted |
| Legumes | Lentils | Boiled |
| Legumes | Lentils | Grilled |
| Legumes | Lentils | Roasted |
| Meat | Beef | Boiled |
| Meat | Beef | Grilled |
| Meat | Beef | Fried |
| Meat | Beef | Roasted |
| Meat | Chicken | Boiled |
| Meat | Chicken | Grilled |
| Meat | Chicken | Fried |
| Meat | Chicken | Roasted |
| Meat | Lamb | Boiled |
| Meat | Lamb | Grilled |
| Meat | Lamb | Fried |
| Meat | Lamb | Roasted |
| Meat | Pork | Boiled |
| Meat | Pork | Grilled |
| Meat | Pork | Fried |
| Meat | Pork | Roasted |
| Tubers | Potatoe | Boiled |
| Tubers | Potatoe | Grilled |
| Tubers | Potatoe | Fried |
| Vegetables | Capsicum | Boiled |
| Vegetables | Capsicum | Grilled |
| Vegetables | Capsicum | Fried |
| Vegetables | Capsicum | Roasted |
| Vegetables | Carrot | Boiled |

| Food Group | Food | Cooking Method |
|---|---|---|
| Vegetables | Carrot | Grilled |
| Vegetables | Carrot | Fried |
| Vegetables | Carrot | Roasted |
| Vegetables | Cauliflower | Boiled |
| Vegetables | Cauliflower | Grilled |
| Vegetables | Cauliflower | Fried |
| Vegetables | Cauliflower | Roasted |
| Vegetables | Onion | Boiled |
| Vegetables | Onion | Grilled |
| Vegetables | Onion | Fried |
| Vegetables | Onion | Roasted |
| Vegetables | Tomatoe | Boiled |
| Vegetables | Tomatoe | Fried |
| Vegetables | Tomatoe | Roasted |

**Table S2.** Correlations between heat damage markers (furosine, HMF and furfural), and the results of antioxidant capacity measured with Folin-Ciocalteu, FRAP and DPPH after digestion and in vitro fermentation processes, depending on the differents foods groups

| CEREALS | M | SD | FUROSINE | HMF | FURFURAL |
|---|---|---|---|---|---|
| Folin-Ciocalteu Digestion | 1935 | 1405 | .41 | .07 | .09 |
| | | | [-.29, .83] | [-.59, .67] | [-.57, .68] |
| Folin-Ciocalteu Fermentatio | 18638 | 5744 | -.49 | .49 | .42 |
| | | | [-.85, .21] | [-.20, .86] | [-.29, .83] |
| FRAP Digestion | 4.12 | 4.29 | .34 | -.25 | -.28 |
| | | | [-.37, .80] | [-.76, .45] | [-.77, .43] |
| FRAP Fermentation | 105 | 17.8 | -.11 | .52 | .37 |
| | | | [-.69, .56] | [-.16, .87] | [-.34, .81] |
| DPPH Digestion | 4.98 | 5.16 | -.64* | -.16 | -.04 |
| | | | [-.91, -.02] | [-.72, .52] | [-.65, .60] |
| DPPH Fermentation | 104 | 23.4 | .12 | -.25 | -.27 |
| | | | [-.55, .70] | [-.76, .45] | [-.77, .43] |
| EGGS | M | SD | FUROSINE | HMF | FURFURAL |
| Folin-Ciocalteu Digestion | 2542 | 1664 | .65 | -.66 | .83 |
| | | | [-.83, .99] | [-.99, .82] | [-.65, 1.00] |
| Folin-Ciocalteu Fermentatio | 49849 | 4956 | .66 | -.51 | .36 |
| | | | [-.82, .99] | [-.99, .88] | [-.92, .98] |
| FRAP Digestion | 5.90 | 0.83 | .41 | -.63 | .47 |
| | | | [-.91, .98] | [-.99, .84] | [-.90, .99] |
| FRAP Fermentation | 282 | 12.0 | .14 | .13 | -.34 |
| | | | [-.95, .97] | [-.95, .97] | [-.98, .92] |
| DPPH Digestion | 9.99 | 0.81 | -.04 | .02 | .77 |
| | | | [-.96, .96] | [-.96, .96] | [-.74, .99] |
| DPPH Fermentation | 235 | 70.6 | .36 | -.41 | .93 |
| | | | [-.92, .98] | [-.98, .91] | [-.31, 1.00] |
| FISH | M | SD | FUROSINE | HMF | FURFURAL |
| Folin-Ciocalteu Digestion | 2626 | 825 | -.02 | .32 | -.53 |
| | | | [-.82, .80] | [-.67, .90] | [-.94, .49] |
| Folin-Ciocalteu Fermentatio | 59530 | 22284 | -.09 | .13 | -.36 |
| | | | [-.84, .78] | [-.76, .85] | [-.91, .64] |
| FRAP Digestion | 2.51 | 0.64 | .43 | .44 | .12 |
| | | | [-.59, .92] | [-.58, .92] | [-.77, .85] |
| FRAP Fermentation | 319 | 130 | -.09 | .13 | -.37 |
| | | | [-.84, .78] | [-.76, .85] | [-.91, .63] |
| DPPH Digestion | 14.2 | 8.95 | .21 | .22 | .07 |
| | | | [-.73, .87] | [-.72, .88] | [-.79, .83] |
| DPPH Fermentation | 214 | 36.7 | -.26 | -.32 | -.12 |
| | | | [-.88, .70] | [-.90, .67] | [-.85, .76] |
| FRUITS | M | SD | FUROSINE | HMF | FURFURAL |
| Folin-Ciocalteu Digestion | 598 | 512 | -.57 | -.64 | -.28 |
| | | | [-.99, .87] | [-.99, .84] | [-.98, .93] |
| Folin-Ciocalteu Fermentatio | 31752 | 8985 | .64 | .63 | .87 |
| | | | [-.83, .99] | [-.84, .99] | [-.56, 1.00] |
| FRAP Digestion | 5.81 | 3.81 | -.55 | -.20 | .24 |
| | | | [-.99, .87] | [-.97, .94] | [-.94, .98] |

| FRUITS | M | SD | FUROSINE | HMF | FURFURAL |
|---|---|---|---|---|---|
| FRAP Fermentation | 126 | 39.2 | -.09 | -.52 | -.96* |
| | | | [-.97, .95] | [-.99, .88] | [-1.00, .04] |
| DPPH Digestion | 22.2 | 33.5 | -.66 | -.63 | -.43 |
| | | | [-.99, .82] | [-.99, .84] | [-.98, .90] |
| DPPH Fermentation | 144 | 24.3 | -.00 | -.99** | -.53 |
| | | | [-.96, .96] | [-1.00, -.69] | [-.99, .88] |
| LEGUMES | M | SD | FUROSINE | HMF | FURFURAL |
| Folin-Ciocalteu Digestion | 1803 | 1532 | .46 | .21 | -.33 |
| | | | [-.71, .95] | [-.83, .92] | [-.94, .78] |
| Folin-Ciocalteu Fermentatio | 43519 | 19159 | .77 | -.23 | -.21 |
| | | | [-.36, .98] | [-.92, .82] | [-.92, .83] |
| FRAP Digestion | 6.95 | 4.52 | -.39 | .17 | -.89* |
| | | | [-.95, .75] | [-.84, .91] | [-.99, -.05] |
| FRAP Fermentation | 240 | 101 | .72 | -.16 | -.33 |
| | | | [-.44, .98] | [-.91, .84] | [-.94, .78] |
| DPPH Digestion | 38.7 | 5.21 | .06 | -.17 | -.78 |
| | | | [-.87, .89] | [-.91, .84] | [-.98, .34] |
| DPPH Fermentation | 187 | 56.3 | -.84 | -.01 | -.70 |
| | | | [-.99, .16] | [-.88, .88] | [-.98, .48] |
| MEAT | M | SD | FUROSINE | HMF | FURFURAL |
| Folin-Ciocalteu Digestion | 2616 | 998 | .00 | -.02 | -.10 |
| | | | [-.51, .52] | [-.53, .50] | [-.59, .43] |
| Folin-Ciocalteu Fermentatio | 49297 | 13682 | -.05 | -.12 | -.12 |
| | | | [-.55, .48] | [-.60, .42] | [-.60, .42] |
| FRAP Digestion | 3.05 | 0.68 | -.58* | .06 | -.00 |
| | | | [-.84, -.10] | [-.47, .56] | [-.51, .51] |
| FRAP Fermentation | 278 | 73.7 | -.17 | -.23 | -.18 |
| | | | [-.63, .37] | [-.67, .32] | [-.64, .36] |
| DPPH Digestion | 14.6 | 6.10 | .48 | .31 | .22 |
| | | | [-.04, .80] | [-.24, .71] | [-.33, .66] |
| DPPH Fermentation | 481.7 | 288 | -.42 | -.38 | -.18 |
| | | | [-.77, .12] | [-.75, .16] | [-.63, .37] |
| VEGETABLES | M | SD | FUROSINE | HMF | FURFURAL |
| Folin-Ciocalteu Digestion | 461 | 394 | .80** | .80** | -.02 |
| | | | [.52, .92] | [.53, .93] | [-.49, .47] |
| Folin-Ciocalteu Fermentatio | 27727 | 13643 | -.08 | -.15 | -.22 |
| | | | [-.54, .42] | [-.59, .36] | [-.64, .29] |
| FRAP Digestion | 5.20 | 5.70 | .80** | .74** | -.04 |
| | | | [.52, .92] | [.40, .90] | [-.51, .45] |
| FRAP Fermentation | 149 | 66.0 | -.04 | -.08 | -.14 |
| | | | [-.51, .45] | [-.54, .42] | [-.58, .36] |
| DPPH Digestion | 17.4 | 20.3 | .84** | .75** | -.03 |
| | | | [.60, .94] | [.42, .90] | [-.50, .46] |
| DPPH Fermentation | 136.3 | 51.4 | -.22 | -.16 | -.08 |
| | | | [-.63, .29] | [-.60, .34] | [-.54, .42] |

Note. SD ais used to represent standard deviation.. Values in square brackets indicate the 95% confidence interval for each correlation. The confidence interval is a plausible range of population correlations that could have caused the sample correlation (Cumming, 2014). * indicates $p < .05$. ** indicates $p < .01$.
